# Increased Synaptic Excitation and Abnormal Dendritic Structure of Prefrontal Cortex Layer V Pyramidal Neurons following Prolonged Binge-Like Consumption of Ethanol

**DOI:** 10.1523/ENEURO.0248-16.2016

**Published:** 2016-12-23

**Authors:** Paul M. Klenowski, Matthew J. Fogarty, Masroor Shariff, Arnauld Belmer, Mark C. Bellingham, Selena E. Bartlett

**Affiliations:** 1 Translational Research Institute and Institute for Health and Biomedical Innovation, Queensland University of Technology, Brisbane 4000, Queensland, Australia; 2 School of Biomedical Sciences, University of Queensland, Brisbane 4072, Queensland, Australia

**Keywords:** dendrite, ethanol, mPFC, pyramidal, spines, synaptic currents

## Abstract

Long-term alcohol use causes a multitude of neurochemical changes in cortical regions that facilitate the transition to dependence. Therefore, we used a model of long-term, binge-like ethanol consumption in rats to determine the effects on morphology and synaptic physiology of medial prefrontal cortex (mPFC) layer V pyramidal neurons. Following 10 weeks of ethanol consumption, we recorded synaptic currents from mPFC neurons and used neurobiotin filling to analyze their morphology. We then compared these data to measurements obtained from age-matched, water-drinking control rats. We found that long-term ethanol consumption caused a significant increase in total dendrite arbor length of mPFC layer V pyramidal neurons. Dendritic restructuring was primarily observed in basal dendrite arbors, with mPFC neurons from animals engaged in long-term ethanol drinking having significantly larger and more complex basal arbors compared with controls. These changes were accompanied by significantly increased total spine densities and spontaneous postsynaptic excitatory current frequency, suggesting that long-term binge-like ethanol consumption enhances basal excitatory synaptic transmission in mPFC layer V pyramidal neurons. Our results provide insights into the morphological and functional changes in mPFC layer V pyramidal neuronal physiology following prolonged exposure to ethanol and support changes in mPFC activity during the development of alcohol dependence.

## Significance Statement

The development of alcohol addiction is a progressive cycle that involves extended periods of heavy alcohol use. Therefore, we investigated the long-term effects of binge-like alcohol consumption on layer V pyramidal neurons in the medial prefrontal cortex (mPFC), a brain region involved in high-order executive processes, including impulsivity. We show that the long-term alcohol consumption increases the dendritic arborization of pyramidal neurons in layer V of the mPFC. These changes were also accompanied by significantly increased spine densities and excitatory synaptic activity in these neurons. Our study also highlights differences between long-term and chronic short-term alcohol intake on mPFC structure and function, adding to the importance of implementing long-term drinking models to better understand neuroadaptations facilitating the transition to dependence.

## Introduction

Long-term alcohol use has significantly different effects on the brain compared with short-term exposure (Koob and Volkow, 2010). Prolonged alcohol use often involves repeated cycles of binge-like intake and abstinence, which produces adaptive changes in the brain that alter decision-making and impulsivity, and that sensitize behavioral responses to negative symptoms of withdrawal (Koob and Volkow, 2010). These effects also produce differential responses to pharmaceutical interventions compared with short-term intake (Steensland et al., 2007; Patkar et al., 2016). Prolonged alcohol use is thought to induce changes in a number of brain circuits and neurochemical pathways that facilitate the transition to dependence. In particular, accumulating evidence suggests that heavy alcohol use alters the activity and structure of the medial prefrontal cortex (mPFC; Holmes et al., 2012; Kroener et al., 2012; Kim et al., 2015).

The mPFC forms part of a complex neural circuit involved in high-order executive processes, including planning, decision-making, and control over impulsivity (Dalley et al., 2004; Crews and Boettiger, 2009; Goldstein and Volkow, 2011). The mPFC sends an organized network of efferent connections to subcortical regions controlling affective and motivated behaviors (Ongür and Price, 2000). Additionally, the mPFC exerts top-down regulation of subcortical sites, including the extended amygdala, which are sensitive to long-term alcohol use (Koob, 2013) and are disrupted during withdrawal (George et al., 2012). Recent studies in rodents have shown that excessive alcohol exposure affects mPFC pyramidal neurons (Holmes et al., 2012; Kroener et al., 2012; Kim et al., 2015). Moreover, results from human imaging studies have shown differences in PFC composition and functionality from alcoholics, including heightened responses to cues associated with alcohol (Grüsser et al., 2004; Myrick et al., 2004). Long-term alcohol consumption may also facilitate PFC-mediated cognitive impairments, leading to an inability to maintain executive control over impulsivity and/or self-limit alcohol intake (Goldstein et al., 2004).

Recent investigations have highlighted the contrast between short-term and long-term ethanol treatment on mPFC pyramidal neuronal activity. Short-term application of ethanol to *ex vivo* brain slices has been shown to cause depolarization of mPFC pyramidal neurons and reduce NMDA-mediated postsynaptic currents (Weitlauf and Woodward, 2008; Woodward and Pava, 2009). Conversely, a longer 3 week regimen of intermittent ethanol vapor [i.e., chronic intermittent ethanol (CIE)] exposure demonstrated increased NMDA-mediated postsynaptic currents and spike timing-dependent plasticity (Kroener et al., 2012). The induction of alcohol dependence with CIE administration has also revealed changes in mPFC pyramidal neuron morphology following short-term exposure (Holmes et al., 2012; Kim et al., 2015). A modest increase in the density of mature spines in basal dendrites (Kroener et al., 2012) and hypertrophy of apical dendrites from layer V pyramidal neurons (LVPNs) were observed following 3–4 weeks of CIE treatment (Holmes et al., 2012). A longer CIE regime (7–10 weeks) revealed a greater level of dendritic hypertrophy and spine density increases in layer 2/3 pyramidal neurons, suggesting that long-term ethanol intake may have a greater effect on mPFC structure compared with short-term exposure (Kim et al., 2015).

While these studies have provided important insights into the effects of chronic ethanol exposure via passive administration, the effects of voluntary ethanol consumption on LVPN morphology and physiology following long-term access remain unknown. Because the choice to voluntarily consume ethanol following prolonged use is thought to result from mPFC dysfunction, which, in part, leads to a loss of control and an inability to self-limit intake, we used an intermittent access, two-bottle choice drinking paradigm in rats (Simms et al., 2008) to determine the effect of long-term ethanol consumption on mPFC LVPN synaptic activity and morphology. This well validated, voluntary access model produces escalating binge-like patterns of ethanol consumption, behavioral intoxication, and withdrawal symptoms during periods of abstinence, all of which are indicators of alcohol dependence (Carnicella et al., 2014). Because recent reports show that pharmaceutical interventions produce differential responses in rodents following short- and long-term binge-like consumption of ethanol (Steensland et al., 2007; Patkar et al., 2016), we gave rats extended access (10 weeks) to ethanol using the voluntary intermittent two-bottle choice model. We then recorded synaptic currents and neurobiotin (NB)-filled mPFC LVPNs in brain slices from long-term ethanol-consuming rats, comparing their synaptic physiology and morphology to LVPNs from age-matched water-drinking controls. We show that long-term, binge-like consumption of ethanol enhances spontaneous EPSC frequency and spine densities, and significantly increases the dendritic arbor length of mPFC LVPNs. These long-term effects suggest that alterations in mPFC LVPN activity and structure are important factors contributing to the development of alcohol dependence.

## Materials and Methods

### Ethics statement

All experimental procedures were approved by The University of Queensland and the Queensland University of Technology Animal Ethics Committees and complied with the policies and regulations regarding animal experimentation and other ethical matters (Drummond, 2009). They were conducted in accordance with the Queensland Government Animal Research Act 2001, associated Animal Care and Protection Regulations (2002 and 2008), as well as the Australian Code for the Care and Use of Animals for Scientific Purposes, 8th Edition (National Health and Medical Research Council, 2013).

### Animals and housing

Five-week-old (adolescent) male Wistar rats (Animal Resource Center) were housed individually in ventilated dual-level Plexiglas cages. The rats were housed in a climate-controlled (22–24°C), 12 h reversed light/dark cycle (lights off at 9:00 A.M.) room and were given access to standard rat chow and water *ad libitum*. All rats were acclimatized to the housing conditions, handling, and reverse light cycle for 1 week prior to the start of treatment.

### Intermittent access two-bottle choice drinking paradigm

We used the intermittent access 20% ethanol two-bottle choice paradigm described in the study by Simms et al. (2008) and adapted from Wise (1973). Briefly, solutions were presented in 300 ml graduated plastic bottles with stainless steel drinking spouts, inserted through two grommets in the front of the cage following commencement of the dark/light cycle. The weight of each bottle was recorded to the nearest 0.1 g prior to presentation. For the ethanol group (*n* = 10), one bottle containing water and a second bottle containing 20% ethanol (v/v) were presented simultaneously. The placement of the 20% ethanol bottle was switched at every presentation to prevent side preference. Bottles were weighed at 30 min, 2 h, and 24 h after presentation, and measurements were taken to the nearest 0.1 g. After 24 h, the ethanol bottle was replaced with a water bottle that was available for the next 24 h. This pattern was repeated three times per week for 10 weeks. The rats had unlimited access to water on all other days. The weight of each rat was also measured to calculate the grams of ethanol intake per kilogram of body weight prior to each ethanol presentation. A separate group of age-matched male control rats (*n* = 10) were given access to water in both bottles (i.e., no ethanol) under the same conditions described above. The mean body weights of control and ethanol-consuming rats following 10 weeks of drinking were 574.0 ± 9.4 and 534.4 ± 20.8 g, respectively. No difference in the increase in total body weight between control and ethanol groups was observed over the drinking period (control group, 359.1 ± 8.9 g; ethanol group, 331.7 ± 20.8 g; *p* = 0.24).

### Blood ethanol concentrations

Blood ethanol concentrations (BECs) were measured after 9 weeks of ethanol consumption. Thirty minutes after the commencement of a standard intermittent access session, the bottles were removed, the rat was anaesthetized, and 70–100 μl of tail blood was collected into an EDTA-coated tube. The blood was centrifuged at 4°C for 20 min at 4000 rpm, and the plasma was separated into aliquots and stored at −80°C until assayed. The nicotinamide adenine dinucleotide-ethanol dehydrogenase spectrophotometric assay was used to measure blood ethanol concentration, as previously described (Zapata et al., 2006; Santos et al., 2013). All reagents used in this assay were purchased from Sigma-Aldrich. BECs were calculated using a standard curve, and all samples and standards were run in triplicate.

### Slice preparations

Following the last drinking session, rats were transferred from the animal facility to the School of Biomedical Sciences, University of Queensland (St. Lucia, QLD, Australia). Rats were transported by vehicle from the animal facility with their usual handler in a transport box with litter from their home cage at the end of their active (dark) cycle. Rats were then kept in a cage with litter from their home cage, over the course of the next light cycle. Rats were given overnight to recover. The next day, the rats were sacrificed by sodium pentobarbitone overdose (60–80 mg/kg, i.p.; Vetcare) and then decapitated. The brain was quickly removed and bathed in ice-cold high-Mg^2+^ Ringer’s solution that contained the following (in mm): 130 NaCl, 3 KCl, 26 NaHCO_3_, 1.25 NaH_2_PO_4_, 5 MgCl_2_, 1 CaCl_2_, and 10 d-glucose (osmolarity, ∼300 mOsm; Vapro 5520 osmometer, Wescor; Bellingham and Berger, 1996). Ringer’s solution was continuously bubbled with 95% O_2_/5% CO_2_ to maintain pH at 7.4. With the aid of a brain atlas (Paxinos and Watson, 2007), coronal sections (300 μm thick) containing the mPFC (2.2–4.2 mm from bregma) were cut using a vibratome (Leica VT 1200S, Leica Biosystems). Cut slices were transferred from ice-cold high-Mg^2+^ Ringer’s solution to the same solution warmed to 34°C in a water bath and incubated for 60 min. The sections were then moved to a normal Ringer’s solution (same as above, except for 1 mm MgCl_2_, 2 mm CaCl_2_) and kept at room temperature (22–24°C) for 30 min prior to the start of recording and labeling.

### Electrophysiology

Patch electrodes were pulled from borosilicate glass capillaries (Vitrex Modulohm, Edwards Medical) giving a tip resistance of ∼3–4 MΩ. The tip of the electrode was filled with 2% NB (Vector Laboratories) in an artificial intracellular solution containing the following (in mm): 135 Cs^+^-methanesulfonate, 6 KCl, 1 EGTA, 2 MgCl_2_, 5 Na-HEPES, 3 ATP-Mg^2+^, 0.3 GTP-Tris, pH 7.25 with KOH, osmolarity of 305 ± 5 mOsm (Kanjhan and Bellingham 2013). The electrode was then backfilled with the same intracellular solution without NB. A single brain slice was placed in a tissue chamber on a Nikon E600FN microscope fitted with infrared differential interference contrast optics, and continuously superfused (1-2 ml/min) with the normal Ringer’s solution at room temperature (22–24°C). The tissue was stabilized with the aid of a metal mesh, and recording electrodes were viewed on a monitor through a 60× water-immersion objective and infrared video camera (Hamamatsu). Recordings and voltage pulse protocols were made with an Axopatch 1D amplifier (Axon Instruments). Data were acquired at a sampling rate of 10 kHz and low-pass filtered at 2 kHz, using PClamp 10.2 software and a Digidata 1332A digitizer (Axon Instruments). Large cell bodies of individual LVPNs within the mPFC (van Aerde and Feldmeyer, 2015) were targeted visually, and a patch-electrode was advanced toward the cell soma using a micromanipulator (MPC-200, Sutter Instrument Company). The electrode tip was placed against the neuronal soma, and then gentle suction was applied until a stable seal of preferably >300 MΩ was obtained.

The semi-loose seal NB electroporation procedure was modified from that described for brainstem motor neurons (Kanjhan and Bellingham, 2013). Square-wave voltage steps (0.5 s, 1 Hz) of 5–25 mV that generated current pulses of 300–500 pA were applied for 6–9 min until rupturing of the membrane occurred. If a tight seal formed (>1 GΩ) during suction through the pipette, the same pulses were applied until membrane rupture, when pulses were stopped and spontaneous EPSCs (sEPSCs) and spontaneous IPSCs (sIPSCs) were recorded at holding membrane potentials of −70 and 0 mV, respectively. Synaptic currents were recorded in 2 min epochs and analyzed off-line using PClamp 10.2 software. Between 100 and 1000 events were analyzed from each cell to determine electrophysiological properties. Input resistance was measured using the slope of the current–voltage curve at holding voltages of 0, −20, and −70 mV, as previously described (van Zundert et al., 2008). The input resistance was measured during the experiment following 4–6 min of recording. Synaptic currents were recorded for a minimum of 12 min, with NB electroporation lasting for a minimum of 3 min, followed by a recovery period of 5–10 min where the NB was allowed to diffuse to the extremities of the dendrites.

### Imaging and morphologic quantification

After filling, sections were left in the bath for a minimum of 5 min to allow the NB to diffuse. The sections were then fixed in 4% paraformaldehyde in 0.1 m PBS, pH 7.4, at room temperature for 30 min. Each section was then washed three times in 0.1 m PBS and transferred into a PBS blocking solution containing 4% bovine serum albumin and 0.5% Triton X-100, then incubated overnight at 4°C. To visualize the NB, sections were incubated for 4 h at 4°C in Cy3-streptavidin (1:500 in blocking solution; Sigma-Aldrich). The labeling quality was determined with a standard fluorescence microscope (BX50, Olympus). Slices were then washed in 0.1 m PBS and mounted on slides (Menzel-Gläser) in glycerol-based *p*-phenylenediamine mounting medium and coverslipped (Menzel-Gläser).

Morphological properties of the mPFC LVPNs filled with NB were analyzed from stacks of confocal images obtained at 63× magnification in a manner similar to those of previous studies (Fogarty et al., 2015; Klenowski et al., 2015). Images containing total dendritic arbors were acquired on a Leica TCS SP8 confocal microscope using a Leica 63×/1.3 numerical aperture glycerol immersion objective with a voxel size of 0.18 × 0.18 × 0.5 µm/pixel (*x*-, *y*-, and *z*-dimensions). Image *z*-stacks of between 100 and 180 images were acquired at a *z*-separation of 0.5 µm. For spine quantification, we obtained images with the same objective at 2.5 zoom. The voxel size for spine images was 0.07 × 0.07 × 0.30 µm/pixel (*x*-, *y*-, and *z*-dimensions). Image *z*-stacks of dendrites with spines contained between 25 and 135 images acquired at a step size of 0.3 µm. Morphological properties (dendritic branching, length, and dendritic spines) and Sholl analysis (Sholl, 1953) of filled cells were performed using Neurolucida software (MBF Bioscience) in a manner identical to previous reports (Fogarty et al., 2015; Klenowski et al., 2015, 2016). For Sholl analysis, the center of concentric spheres was defined as the center of the soma, and a 20 µm radius interval was used. All neurons included for analysis showed high-quality filling and contained an intact basal or apical (or both) dendritic arbors. Only large-bodied neurons that were a minimum of 400 µm away from the pia and/or contained an apical dendrite pointing toward the pia were included in the analysis (Gabbott et al., 1997; van Aerde and Feldmeyer, 2015). An entire arbor consisted of the entirety of the length of the dendritic trees emanating from the neuronal soma. A dendritic tree consisted of all of the branches emanating from a single primary (first-order) branch extending from the neuronal soma (Larkman and Mason, 1990). Dendritic processes were classified as spines only if their length was not >6 µm (Harris and Kater, 1994). All neuronal tracing and analysis of synaptic currents was conducted in a blinded fashion.

### Statistical methods

The mean and SEM were calculated for each dataset. Where indicated, unpaired Student’s two-tailed *t* tests or two-way ANOVAs with Bonferroni post-tests were conducted for all analyses involving the comparison of group means. The Kolmogorov–Smirnov test was used to compare cumulative frequency distributions. Correlations were performed with Pearson coefficients. All analyses were performed using Prism 6 (GraphPad). Statistical significance was accepted at *p* < 0.05, and values are reported to two significant figures in text and tables. All data in the Results section are presented as the mean ± SEM.

## Results

### Rats given access to ethanol showed binge-like ethanol consumption and increased preference for ethanol over water

**Figure 1.**
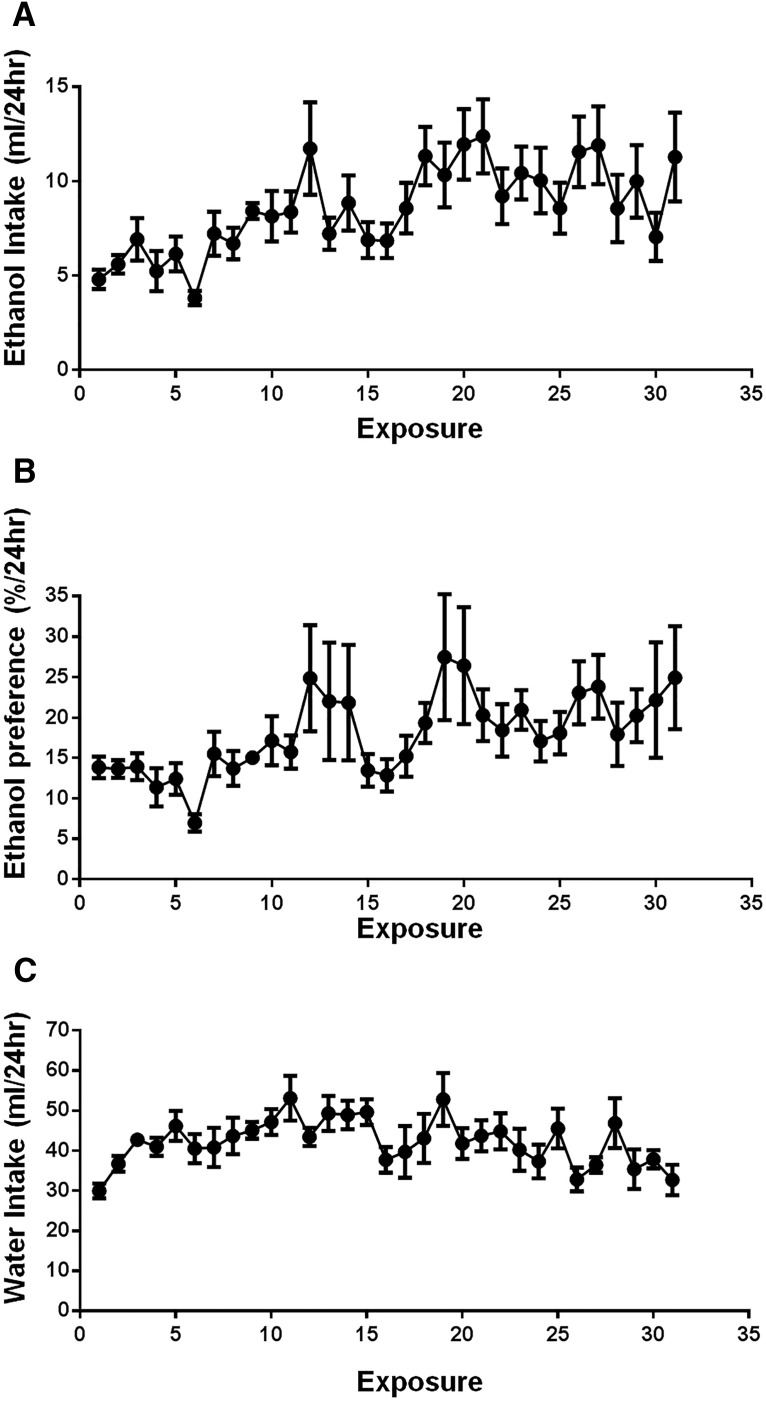
Intermittent access to ethanol facilitates binge patterns of consumption. ***A***, ***B***, Rats (*n* = 10) given intermittent access to ethanol escalated their total intake in a binge-like manner (***A***) and increased their preference for ethanol (***B***) during the drinking period. Following 9 weeks of prior ethanol consumption (∼27 exposures), rats consumed on average 2.10 ± 0.40 g/kg and reached blood ethanol concentrations of 0.09 ± 0.01 g/dl during the first 30 min of ethanol presentation.

Rats given intermittent access to ethanol escalated their total intake by 58% over the exposure period (Fig. 1*A*). Additionally, the intermittent two-bottle choice model facilitated a 44% increase in the preference for ethanol over water during the drinking sessions (Fig. 1*B*). Water consumption over the ethanol exposure period is shown in Figure 1*C*. Following 9 weeks of exposure, this drinking behavior resulted in a mean consumption of 2.10 ± 0.40 g/kg during the first 30 min of ethanol presentation, which produced mean BECs of 0.09 ± 0.01 g/dl (*n* = 9). This corresponds to an average blood alcohol concentration observed in humans after six standard drinks consumed in <2 h.

### Long-term ethanol consumption caused increased excitatory synaptic neurotransmission and neuronal input resistance in mPFC LVPNs

**Figure 2.**
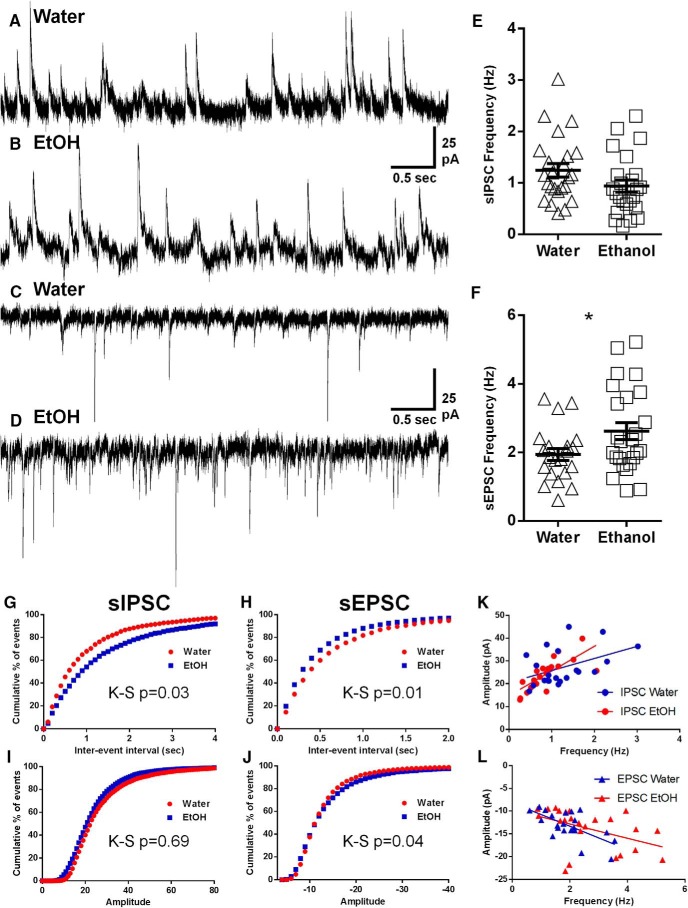
Increased EPSC synaptic drive on LVPNs from the mPFC in ethanol-consuming rats compared with water-consuming controls. Voltage-clamp whole-cell recordings showing an increase in the spontaneous EPSC frequency recorded from mPFC LVPNs in long-term ethanol-drinking rats compared with age-matched water-drinking controls at 16 weeks. ***A–D***, Representative spontaneous IPSCs (upward deflections in ***A*** and ***B***) and spontaneous EPSCs (downward deflections in ***C*** and ***D***) recorded from mPFC LVPNs of control (***A***, ***C***) and ethanol-consuming rats (***B***, ***D***) are shown. ***E***, ***F***, Scatterplots with mean ± SEM show unchanged spontaneous IPSC (***E***, *n* = 21**)** and increased spontaneous EPSC (***F***, *n* = 25, Unpaired two-tailed Student's *t* test, *p < 0.05) frequencies. Calibration: 25 pA, 500 ms. ***G–J***, Cumulative frequency distributions of sIPSC and sEPSC interevent intervals and amplitudes are shown in ***G***–***J*,** respectively, with sIPSC interevent interval (***G***), sEPSC interevent interval (***H***), and sEPSC amplitude (***J***) distributions being significantly different (Kolmogorov–Smirnov test). ***K***, ***L***, Correlation *x–y* plots and linear regressions of sIPSC amplitude against frequency (***K***) and sEPSC amplitude against frequency (***L***), with neither the slope nor the elevations of the regression being significantly different in either case between ethanol-consuming and water-consuming control groups.

To determine the effects of long-term, binge-like ethanol consumption on functional synaptic activity, we analyzed sEPSCs and sIPSCs recorded from mPFC LVPNs obtained from rats consuming water or ethanol (Table 1). The sEPSC frequency was significantly increased by 35% in rats consuming ethanol compared with water-consuming controls (*p = 0.04; Table 1; Fig. 2*C*,*D*,*F*). Long-term ethanol consumption had no effect on sEPSC amplitude, rise time, or half-width (Table 1). Comparison of sIPSC frequency, amplitude, and half-width revealed no difference between the two groups; however, we did observe a 29% increase in rise time in ethanol-consuming rats compared with controls (*p = 0.03; Table 1). Additionally, the cumulative frequency distributions for the interevent interval of both the sIPSCs (Fig. 2*G*) and sEPSCs (Fig. 2*H*) were significantly different between the ethanol and water groups (*p = 0.03, sIPSC; *p = 0.01, sEPSC). The cumulative frequency distribution of sEPSC amplitude was also significantly different between the groups (*p = 0.04; Fig. 2*J*). To further assess whether amplitudes have any effect on the likelihood of current detection, we plotted sIPSC amplitudes against sIPSC frequency (Fig. 2*I*), sEPSC amplitude against sEPSC frequency (Fig. 2*J*), and compared the respective linear regressions. For both sIPSCs and sEPSCs, there was no difference between linear regressions in the ethanol and water control groups (*p* = 0.13, sIPSC slope; *p* = 0.92, sIPSC elevation; *p* = 0.37, sEPSC slope; *p* = 0.58, sEPSC elevation; Fig. 2*K*,*L*).

**Table 1:** Spontaneous EPSCs, IPSCs, and intrinsic parameters of LVPNs within the mPFC

Parameter	Water-consuming controls (*n*)	Ethanol-consuming rats (*n*)	*p* value
sEPSC frequency (Hz)	1.9 ± 0.2 (21)	2.6 ± 0.3 (25)	0.04*
sEPSC amplitude (pA)	−13.2 ± 0.7 (21)	−13.9 ± 0.9 (25)	0.49
sEPSC rise time (ms)	2.7 ± 0.1 (21)	2.6 ± 0.1 (25)	0.44
sEPSC half-width (ms)	3.0 ± 0.2 (21)	3.1 ± 0.1 (25)	0.82
sIPSC frequency (Hz)	1.2 ± 0.1 (24)	0.9 ± 0.1 (24)	0.09
sIPSC amplitude (pA)	26.6 ± 1.6 (24)	24.4 ± 1.4 (24)	0.31
sIPSC rise time (ms)	4.2 ± 0.34 (24)	5.5 ± 0.5 (24)	0.03*
sIPSC half-width (ms)	9.7 ± 0.7 (24)	11.0 ± 0.8 (24)	0.24
Input resistance (MΩ)	206 ± 27 (16)	269 ± 18 (24)	0.047*

All data presented as the mean ± SEM.

*p < 0.05, unpaired two-tailed Student’s *t* test.

Intrinsic neuronal excitability of mPFC LVPNs was also altered by long-term ethanol consumption. Neuron input resistance significantly increased by 31% for mPFC pyramidal neurons from ethanol-drinking rats, compared with water-consuming controls (*p < 0.05; Table 1). As this could influence our electrophysiological and morphological measurements, we assessed correlations between synaptic currents and dendritic properties. We observed no correlation between input resistance and EPSC frequencies in either control (*r* ^2^ = 0.0066, *p* = 0.79) or ethanol-treated (*r* ^2^ = 0.0007, *p* = 0.90) rats. Similarly, there were no correlations between input resistance and dendritic spine density in either control (*r* ^2^ = 0.0127, *p* = 0.74) or ethanol-treated (*r* ^2^ = 0.0177, *p* = 0.57) rats. Together, these results indicate that there is minimal confounding of our measurements between the control and ethanol populations of neurons with differing input resistance.

### Increased total dendritic arbor and basal dendritic arbor length in mPFC LVPNs of ethanol-consuming rats

**Figure 3.**
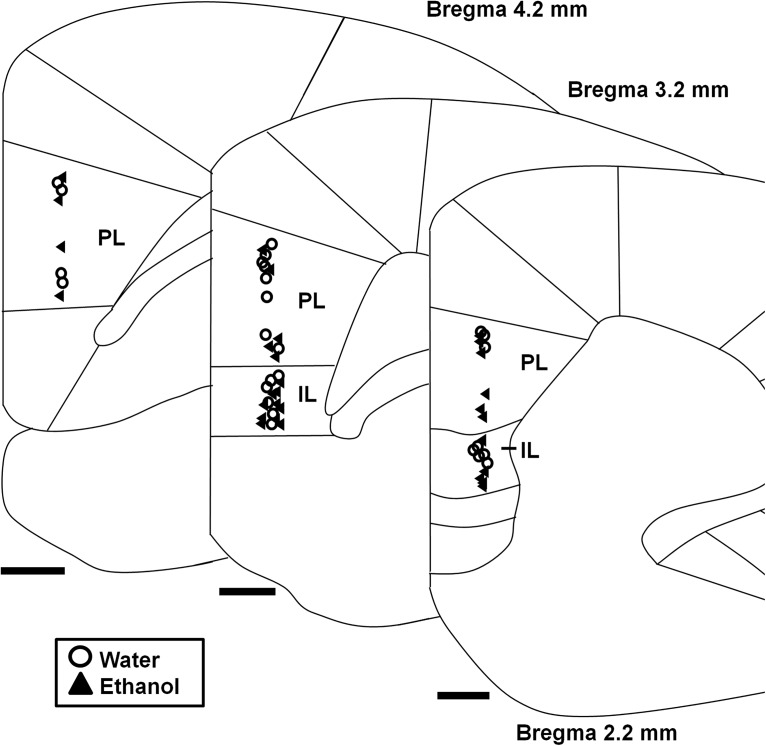
Cortical cytoarchitecture map showing locations of recovered mPFC LVPNs labeled with neurobiotin. Diagram shows the medial portion of the coronally sectioned cortex (2.2–4.2 mm from bregma), with demarcation of the prelimbic (PL) and infralimbic (IL) divisions of the mPFC outlined. The location of neurons from water-consuming control (circles, *n* = 27) and ethanol-consuming (triangles, *n* = 26) rats used for morphological assessment is evenly distributed both rostrocaudally and in the PL and IL regions. Scale bars, 500 μm.

**Figure 4.**
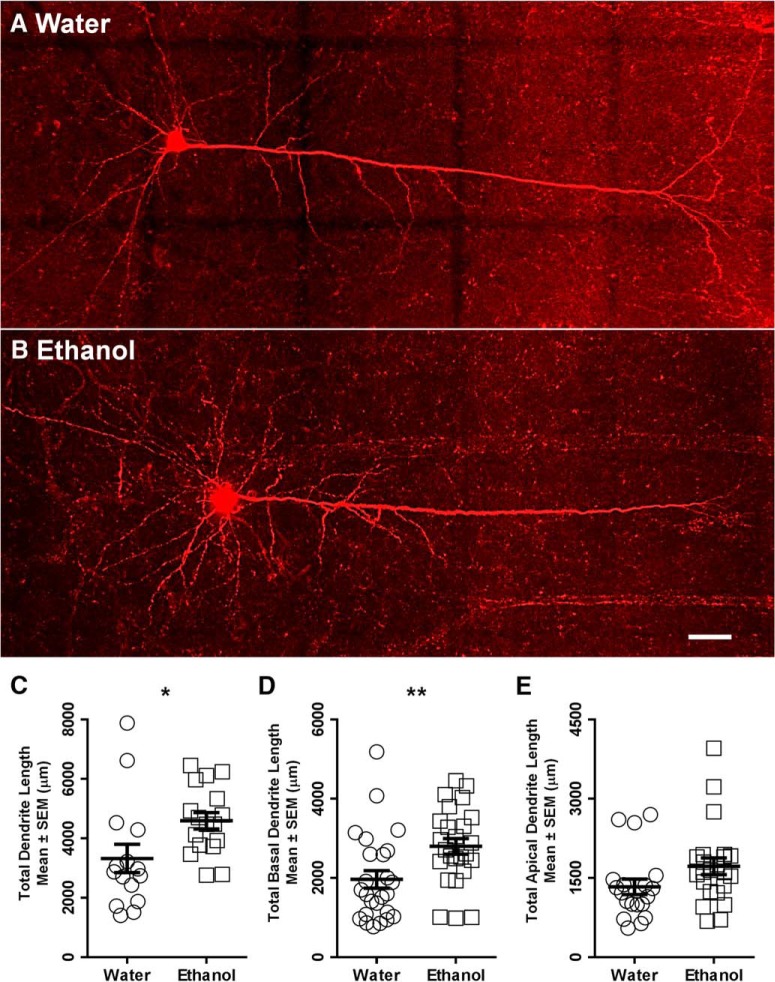
Increased total and basal dendritic arbors of LVPNs from the mPFC of ethanol-consuming rats compared with water-consuming controls. ***A***, ***B***, Images show 40× objective mosaics of mPFC LVPNs from water-consuming control rats (***A***) and ethanol-consuming rats (***B***). ***C–E***, Scatterplots with mean ± SEM of total dendritic length (water, *n* = 15; ethanol, *n* = 18), basal dendritic length (water, *n* = 25; ethanol, *n* = 25), and apical dendritic length (water, *n* = 19; ethanol, *n* = 23) showing significant increases in ethanol-consuming rats in ***C*** and ***D***, but not in ***E***. Unpaired two-tailed Student’s *t* tests, *p < 0.05, **p < 0.01. Scale bar, 50 μm.

**Figure 5.**
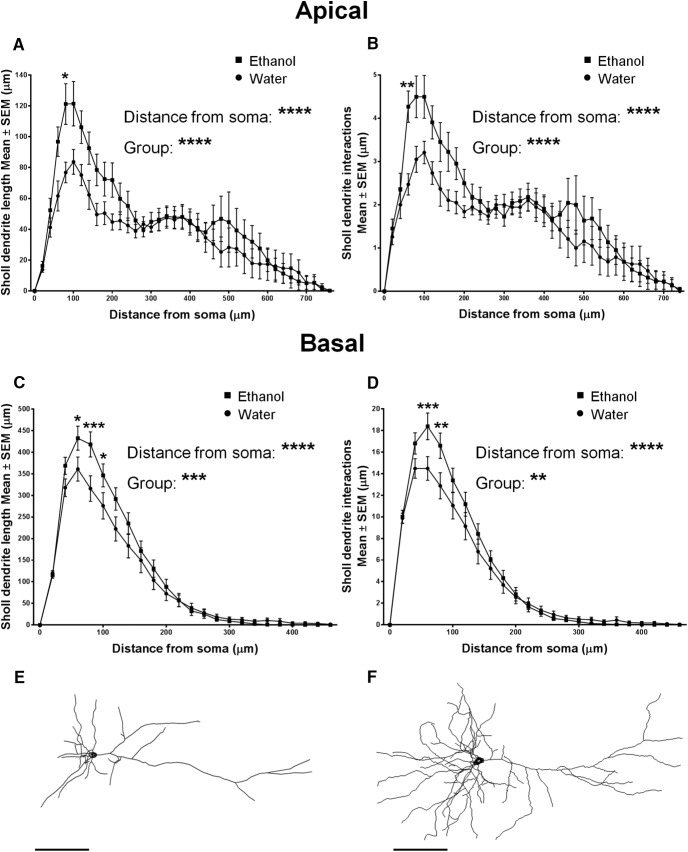
Sholl analysis shows increased length and branching of apical and basal dendrites from mPFC LVPNs of ethanol-consuming rats compared with water-consuming controls. ***A–D***, Sholl analysis of mPFC LVPNs from ethanol- and water-consuming animals revealed a significant effect of ethanol on the length and branching of apical dendrites (***A***, ***B***: water, *n* = 19; ethanol, *n* = 23) and basal dendrites (***C***, ***D***: water, *n* = 25; ethanol, *n* = 25). An increased number of interactions and increased length at apical dendrites 40–60 µm and 60–80 µm away from the soma, respectively, was observed in ethanol consumers compared with controls. For basal dendrites, increased branching and length were found over a range of dendrites that were between 40 and 100 µm away from the soma following long-term ethanol consumption. ***E***, ***F***, Representative traces of mPFC LVPNs from water-consuming (***E***) and ethanol-consuming (***F***) groups. Two-way ANOVAs with Bonferroni post-tests, *p < 0.05, **p < 0.01, ***p < 0.001, ****p < 0.0001. Scale bars, 100 μm.

A persistent increase in the excitatory synaptic drive onto mPFC LVPNs and their input resistance is likely to relate to changes in the somatodendritic surface area and volume of individual mPFC LVPNs after ethanol consumption (van Zundert et al., 2008; Fogarty et al., 2016). We therefore investigated the dendritic morphology of the mPFC LVPNs in response to these altered synaptic inputs. LVPNs from the prelimbic and infralimbic subdivisions of the mPFC (Fig. 3) were filled with NB and were pooled together for analysis (Fig. 3). There was no difference in neuronal soma volume (calculated using volume of an ellipsoid) between the ethanol- and water-consuming groups (*p* = 0.41; Table 2). The total dendritic arbor length of mPFC LVPN dendrites increased by 41% in ethanol consumers, compared with water-consuming controls (*p = 0.02; Table 2; Fig. 4*C*). Comparison of the apical arbors of LVPNs between the groups showed no significant change in the dendritic length (*p* = 0.08; Table 2; Fig. 4*E*) or maximum apical reach, as measured by the distance from the soma to the longest apical dendritic termination at the pia (*p* = 0.62; Table 2). Conversely, the total basal dendritic arbor length of LVPNs was increased by 42% in ethanol-consuming rats compared with controls (**p < 0.01; Table 2; Fig. 4*D*), and the mean basal tree arbor length of LVPNs from the ethanol-consuming group was increased by 44% compared with water-consuming controls (**p < 0.01; Table 2). Sholl analysis showed a significant increase in dendritic length and the number of interactions in both the apical (****p < 0.0001; Fig. 5*A*,*B*) and basal (length: Fig. 5*C*, ***p = 0.0002; interactions: Fig. 5*D*, **p = 0.001) dendritic arbors from ethanol-consuming rats compared with controls. Post-tests revealed that apical dendrites 60–80 µm from the soma were longer (*p = 0.01) and exhibited more interactions between 40 and 60 µm (**p = 0.007) in the ethanol-consuming group compared with the control group. For basal arbors, increased length in the ethanol-consuming group compared with controls was observed in dendrites between 40 and 100 µm from the soma (40–60 µm, *p = 0.04; 60–80 µm, ***p = 0.0002; 80–100 µm, *p = 0.04). Basal dendrites that were 40–80 µm from the soma exhibited more intersections in the ethanol-consuming rats compared with the water-consuming controls (40–60 µm, ***p = 0.0007; 60–80 µm, **p = 0.002).

**Table 2:** General morphologic parameters of LVPNs within the mPFC

Parameter	Water-consuming controls (*n*)	Ethanol-consuming rats (*n*)	*p* value
Soma volume (μm^3^)	1117 ± 112 (27)	1247 ± 109 (26)	0.41
Total dendrite length (μm)	3323 ± 476 (15)	4672 ± 275 (18)	0.02*
Mean apical tree length (μm)	1332 ± 146 (19)	1723 ± 158 (23)	0.08
Max apical terminal length (μm)	521 ± 29 (19)	541 ± 29 (23)	0.62
Total basal tree length (μm)	1965 ± 222 (25)	2798 ± 195 (25)	0.007**
Mean basal tree length (μm)	334 ± 40 (25)	481 ± 33 (25)	0.007**

All data are presented as the mean ± SEM.

*p < 0.05, unpaired two-tailed Student’s *t* test.

**p < 0.01, unpaired two-tailed Student’s *t* test.

**Figure 6.**
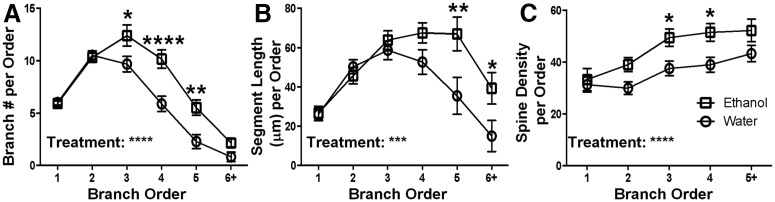
Basal dendrites show increased branch length and branch number, and increased spine density for distal branch orders in LVPNs from the mPFC of ethanol-consuming rats compared with water-consuming controls. ***A***, A plot of the mean branch number per centrifugal branch order for basal dendrites, with significant increases for third-, fourth-, and fifth-order branches (*n* = 25). ***B***, A plot of the mean segment length (in micrometers) per centrifugal branch order for basal dendrites, with increased segment length for fifth-order and sixth-order or greater branches (*n* = 25). ***C***, A plot of the mean spine density (spines per 100 μm length) per centrifugal branch order (*n* = 25). Two-way ANOVAs with Bonferroni post-tests, *p < 0.05, **p < 0.01, ***p < 0.001, ****p < 0.0001.

Following the characterization of the gross morphology of mPFC LVPNs, we further analyzed the branch-order characteristics of their basal dendritic arbors, by quantifying the number of dendritic segments per branch order and the mean length of dendritic segments per branch order (Fig. 6). We found an increased number of basal dendritic segments per branch order in the ethanol-consuming group compared with the control group (Fig. 6*A*, ****p < 0.0001), with post-tests showing significant increases at third (*p = 0.01), fourth (****p < 0.0001), and fifth (**p = 0.002) branch orders. In addition, we found an increase in the mean length of basal dendritic segments per branch order between the ethanol-consuming and control groups (Fig. 6*B*, ***p = 0.0008), with post-tests showing significant increases at fifth (**p = 0.002) and sixth or greater (*p = 0.03) branch orders. These data show that basal dendrite hypertrophy after long-term ethanol consumption is selectively targeted to tertiary or higher branch orders.

### Increased total apical and basal spine density of mPFC LVPNs in ethanol-consuming rats compared with water-consuming controls

The increases in sEPSC frequency and in basal dendrite length could alter the number of presynaptic inputs onto the LVPNs in the mPFC. As dendritic spines are the morphological correlate of excitatory synapses in pyramidal neurons (Spruston, 2008), we quantified the dendritic spine densities of mPFC LVPNs from ethanol- and water-consuming groups.

**Figure 7.**
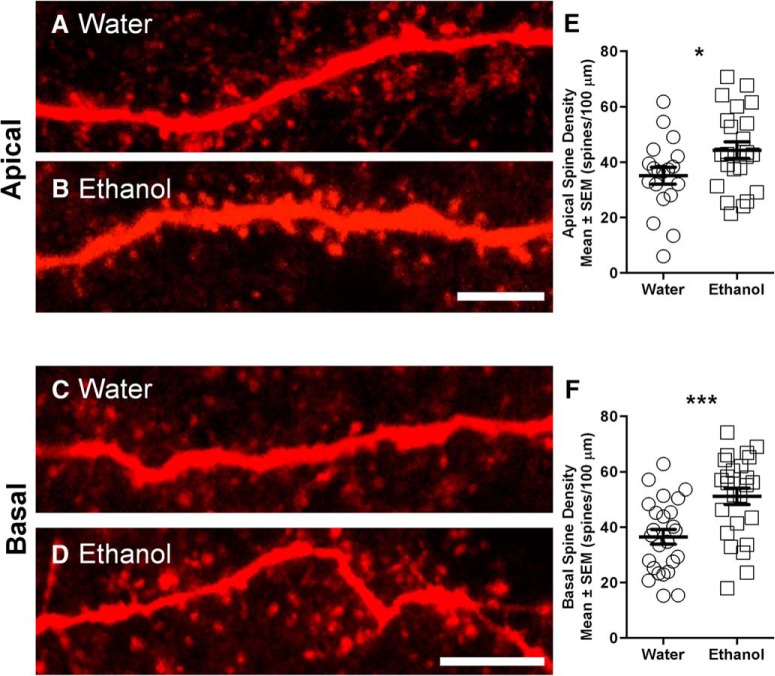
Increased dendritic spine density in both apical and basal dendrites of mPFC LVPNs from ethanol-consuming rats compared with water-consuming controls. ***A***, ***B***, High-magnification images of apical dendritic segments of mPFC LVPNs from water-consuming controls (***A***) and ethanol-consuming rats (***B***). ***C***, ***D***, High-magnification images of basal dendritic segments of mPFC LVPNs from water-consuming controls (***A***) and ethanol-consuming rats (***B***). ***E***, ***F***, Scatterplots with mean ± SEM of total spine density per 100 μm of apical dendrite (water, *n* = 19; ethanol, *n* = 23) and basal dendrite (water, *n* = 25; ethanol, *n* = 25), respectively. Unpaired two tailed Student’s *t* tests, *p < 0.05, ***p < 0.001. Scale bars, 5 μm.

The overall spine density per 100 μm of dendrite increased by 29% in the ethanol-consuming group compared with the control group (*p = 0.03; Table 3). By contrast with the selective increase in basal dendrite length and complexity, changes in spine density were seen in both apical and basal dendrites. The spine density per 100 μm of apical dendrite increased by 26% in ethanol consumers compared with water-consuming controls (*p = 0.04; Table 3; Fig. 7*E*). The basal dendrite spine density increased by 40% in LVPNs of ethanol-consuming rats compared with water-consuming controls (***p < 0.001; Table 3; Fig. 7*F*). Branch order analysis of basal dendrites showed a significant effect of ethanol consumption on spine density per branch order (***p < 0.001; Fig. 6*C*), with post-tests revealing significant increases at third (*p = 0.04) and fourth (*p = 0.03) branch orders. Together, our spine density data show that a significant level of structural plasticity is induced in mPFC LVPNs by extended periods of binge-like ethanol consumption.

**Table 3:** Spine density (spines per 100 μm of dendrite) parameters of LVPNs within the mPFC

Parameter	Water-consuming controls (*n*)	Ethanol-consuming rats (*n*)	*p* value
Total dendrite density	37.7 ± 3.4 (15)	48.6 ± 3.4 (18)	0.03*
Total apical density	35.2 ± 3.1 (19)	44.4 ± 3.0 (23)	0.04*
Total basal density	36.5 ± 2.6 (25)	51.2 ± 3.0 (25)	0.0006***

All data are presented as the mean ± SEM.

*p < 0.05, unpaired two-tailed Student’s *t* test.

***p < 0.001, unpaired two-tailed Student’s *t* test.

## Discussion

In this study, we have used a combination of behavioral, electrophysiological, and imaging techniques to provide a detailed analysis of mPFC LVPNs from long-term ethanol drinking and age-matched water-consuming control rats. Using a voluntary intermittent two-bottle choice drinking paradigm that facilitated binge-like ethanol consumption, increased ethanol preference, and clinically relevant blood ethanol concentrations, we have been able to demonstrate for the first time the long-term effects of heavy alcohol consumption on mPFC LVPN morphology and physiology. Our analysis of postsynaptic currents revealed that long-term ethanol consumption induced a significant enhancement of sEPSC frequency in mPFC LVPNs. Long-term ethanol intake also induced considerable changes in intrinsic and morphological neuronal properties. Higher neuronal input resistance was accompanied by an increase in total dendritic arbor length and increased total, apical, and basal spine densities in mPFC LVPNs from long-term ethanol-consuming rats compared with controls. Our Sholl and branch-order analyses showed that dendritic restructuring induced by long-term ethanol consumption was primarily confined to the more distal dendrite branches that were between 40 and 100 µm away from the soma. These results provide further evidence to support an association between prolonged ethanol consumption and mPFC dysfunction.

In humans, long-term alcohol abuse is thought to cause adaptive changes in the brain that facilitate a behavioral state of dependence, which is characterized by compulsive drinking, an inability to self-limit intake and the development of a negative emotional state during withdrawal (Koob and Volkow, 2010). Additionally, repeated cycles of binge drinking and abstinence are thought to play a key role in the development of alcohol dependence (Koob, 2013). Despite these observations, few studies have investigated the long-term effects of voluntary alcohol consumption, particularly at the cellular and synaptic levels. Not surprisingly, such long-term studies present technical challenges, particularly with regard to obtaining electrophysiology recordings in aged rodents. Because we have previously found that the length of alcohol consumption is an important factor driving the adaptive changes to alcohol (Steensland et al., 2007; Feduccia et al., 2014; Patkar et al., 2016), we implemented NB filling using a semi-loose seal (Klenowski et al., 2015) and electroporation method (Kanjhan and Bellingham, 2013), which has allowed us to obtain an adequate level of cell recovery and stable whole-cell configurations in mPFC LVPNs from 16-week-old rats, following intermittent access to ethanol for 10 weeks. Importantly, this exposure period is similar to previous work from our group, which has demonstrated differential pharmacological and neuronal effects following short-term (4 weeks) and long-term (10–12 weeks) consumption of ethanol (Steensland et al., 2007; Feduccia et al., 2014; Patkar et al., 2016) and sucrose (Klenowski et al., 2016; Shariff et al., 2016).

Previous studies have highlighted the acute and chronic short-term effects of ethanol on the mPFC. Short-term application of ethanol to rat brain slices decreased NMDA-mediated EPSC activity of deep-layer prelimbic cortex pyramidal neurons and inhibited persistent activity during up-states (Tu et al., 2007; Weitlauf and Woodward, 2008). Conversely, chronic short-term ethanol treatment via vapor inhalation was shown to enhance excitatory activity of deep-layer mPFC pyramidal neurons by increasing NMDA-mediated, spike timing-dependent plasticity and the evoked NMDA/AMPA synaptic current ratio (Kroener et al., 2012). The effects of short-term vapor inhalation also changed the morphology of mPFC pyramidal neurons by inducing apical dendrite hypertrophy and increased spine density (Holmes et al., 2012; Kroener et al., 2012). Moreover, 4 d binge ethanol consumption treatment has been shown to modulate effectors of neurogenesis and to promote necrosis in the frontal cortex (Obernier et al., 2002; Crews and Nixon, 2009).

These studies have provided important insights into the sensitivity of the mPFC to acute or chronic short-term ethanol intake. Importantly, our study focused on long-term ethanol-induced changes in mPFC LVPN structure and function, as long-term alcohol consumption produces additional responses that are critically involved in the development of alcohol dependence and that are lacking in short-term use. Additionally, we used the voluntary intermittent access two-bottle choice drinking model, which allows rats to choose whether or not to consume ethanol and how much ethanol they consume, unlike passive ethanol vapor administration, which is typically associated with higher BECs.

Results from human imaging studies have demonstrated reductions in PFC gray matter among drug-addicted subjects, including those addicted to alcohol (Fein et al., 2002; Chanraud et al., 2007). These changes are thought to contribute to cognitive deficits and impairment of executive functions that together facilitate compulsive drinking behavior (Goldstein et al., 2004; Goldstein and Volkow, 2011). However, the direct contribution of long-term alcohol use to these effects is difficult to interpret and can be hard to pin down, as human alcoholic individuals often present with various comorbidities that could influence changes in PFC composition.

Recent animal studies have begun to uncover the long-term effects of ethanol on the mPFC. Using a similar intermittent access two-bottle choice drinking paradigm, 6 weeks of ethanol consumption was shown to increase neuronal activity in the mPFC during withdrawal, as determined using global measurements of Fos expression (George et al., 2012). Additionally, extended CIE treatment for up to 10 weeks caused a significant increase in the dendritic branching and spine densities of basal and apical dendrites, but decreased the dendritic reach of layer II/III neurons (Kim et al., 2015). Our morphology data for LVPNs has revealed that long-term ethanol consumption causes a substantially greater level of dendritic changes compared with previous studies (Holmes et al., 2012; Kroener et al., 2012; Kim et al., 2015). Not only did mPFC LVPNs from long-term ethanol consuming rats show an increase in the total dendritic length, but significant lengthening and more complex branching patterns in basal dendrites were also observed. Our comprehensive assessment of basal dendrite branch-order architecture also pinpointed a significant increase in the number of third-, fourth-, and fifth-order branches and an increase in the mean length of fifth and sixth or greater branch orders in mPFC LVPNs from ethanol-consuming rats, compared with water-consuming controls. While long-term ethanol consumption caused less change in the gross morphology of apical dendrites, Sholl analysis did reveal a significant effect of long-term ethanol consumption on length and dendritic branching, similar to the level of dendritic restructuring observed in previous reports (Holmes et al., 2012; Kim et al., 2015). Although stress associated with the transport of the rats may have in part contributed to the observed differences, all animals in this study underwent the same transport protocol and were provided with a stable environment and time to acclimatize to the new environment in order to reduce this potential confound.

How these alterations affect the signal integration of synaptic inputs to mPFC following voluntary long-term ethanol consumption requires further investigation. Impaired neuronal processing may be due to increased attenuation of excitatory postsynaptic potential in the lengthened dendrites (Stuart and Spruston, 1998). Indeed, our dendritic and dendritic spine changes observed in voluntary ethanol-consuming rats may result in altered spatial and temporal summations (Williams and Stuart, 2002, 2003), perturbing action potential propagation to the axon initial segment and thus contributing to changed firing patterns from the mPFC to target structures (Spruston, 2008).

We found that mPFC LVPNs from long-term ethanol-consuming rats showed increases in the total, apical, and basal spine densities. In both the ethanol-consuming and water-consuming control groups, our quantification included examples of the full array of conventional dendritic spine classifications, including thin, stubby, and mushroom (Jones and Powell, 1969; Peters and Kaiserman-Abramof, 1970). Limitations in our confocal light microscopy (Harris et al., 1992; Arellano et al., 2007; Maiti et al., 2015) and the large variability and prevalence of intermediate spine forms precluded unequivocal categorization of our samples. Indeed, past reports argue that spines exist as a continuum of structures rather than as discrete units (Peters and Kaiserman-Abramof, 1970; Harris et al., 1992; Maiti et al., 2015). In addition to our dendritic spine density changes, increased sEPSC frequency and a reduction in the cumulative probability of the sIPSC interevent interval was also observed in mPFC LVPNs from ethanol-consuming rats compared with controls. These data are in line with previous observations showing enhancement of excitatory synaptic inputs, including NMDA-mediated increases in neuronal excitability and increased NMDAR expression in deep-layer mPFC neurons following chronic ethanol vapor treatment (Kroener et al., 2012). LVPNs within the mPFC receive diverse inputs from a number of brain regions and local neurochemical modulators, including corticotrophin-releasing factor and acetylcholine, which are sensitive to alcohol and could thus influence the excitatory drive onto these neurons. In particular, hyperactivity of the limbic inputs to the mPFC are also thought to underlie the behavioral effects of compulsive drug seeking (Bechara, 2005) and to play a role in the extinction of alcohol addiction (Gass et al., 2014). Previous studies have also identified alcohol-induced dysfunction of cortico–amygdala circuits following CIE and binge-like ethanol consumption (George et al., 2012; Pleil et al., 2015). Furthermore, human brain imaging has revealed decreased dopamine responses (Volkow et al., 2007) and reduced dopamine transmission in the mPFC of alcohol-dependent subjects (Narendran et al., 2014).

In conclusion, our study is the first to demonstrate the long-term effects of binge-like ethanol consumption on mPFC LVPN activity and structure, using a voluntary intermittent two-bottle choice drinking paradigm. Our results highlight differences with respect to changes caused by passive ethanol administration using vapor inhalation models. Our data support converging evidence from human brain imaging and animal studies that have implicated mPFC dysfunction as an important contributor to compulsive alcohol seeking and the development of alcohol dependence. Our study also suggests that reducing excitatory drive onto mPFC LVPNs may represent a novel strategy for managing alcohol consumption following prolonged use.

## Synthesis

Reviewing Editor: Alfonso Araque, University of Minnesota Decisions are customarily a result of the Reviewing Editor and the peer reviewers coming together and discussing their recommendations until a consensus is reached. When revisions are invited, a fact-based synthesis statement explaining their decision and outlining what is needed to prepare a revision will be listed below. The following reviewer(s) agreed to reveal their identity: L. Chandler, Marco Fuenzalida.

The reviewers have found merit and interest on the findings reported. I concur with the reviewers' comments expressing the interest of the study. However, they also expressed some concerns about several issues that need to be addressed and clarified. I also consider pertinent the concerns expressed. I believe that addressing those concerns, which will definitely improve the manuscript, is necessarily required to firmly validate the results.

Specific comments of the reviewers:

Reviewer 1.

General comment:

This is a reasonably interesting study that examined changes in the electrophysiological and morphological properties of layer V pyramidal neurons associated with voluntary chronic intermittent ethanol drinking in rats. While the results are largely confirmatory of previous studies and do not break new ground, the extension and confirmation of these changes in response to voluntary ethanol consumption is an important new observation.

Specific comments:

While the replication of the effect of chronic ethanol exposure using the two-bottle drinking is and important aspect of the present study, this aspect needs to be made more forcefully articulated in the manuscript. In particular, the manuscript would benefit by directly stating that previous studies (most of which were cited in the manuscript) examined changes in response to chronic alcohol in exposure models that involve passive ethanol administration that are typically associated with much higher BECs.

There is some concern that the population of neurons recorded from in the controls and ethanol exposed rats are different since the neurons from the ethanol exposed rats have a higher input resistance. I understand that this difference may actually reflect the effect of ethanol. The larger sized cells my also account/contribute to the dendritic morphological differences. It might be informative to compare the electrophysiological and morphological results in neurons with the same input resistance from the control and ethanol treated rats.

There is some confusion/concern regarding the presentation of the drinking data as ml/24 hrs. It does not appear that the changes in body weight was taken into consideration and factored into the analysis. It appears that drinking was initiated around PD 42 (~6wks), which is ~ middle adolescence and the body weights were likely somewhere around 150 g (I'm guessing). Yet at the end of drinking, the weight was reported to be >500g. How was this accounted for in the calculation of ethanol consumption?

I agree with the concern raised by the previous reviewer regarding the confound of transfer of animals from one institution to another. There needs to be a bit more information provided in regards to how the physical/logistic aspects of the transfer and over what period of time. In addition, it needs to be stated in the discussion that this represents a potential confound of the results.

Reviewer 2.

The authors provide a good description of layer 5 pyramidal cell dendrites and synaptic input in long-term ethanol drinking. The results support the notion that alcohol consumed over long periods can affect the morphology and synaptic function. However, the results and conclusions need to be strengthened.

Changes in the morphology of dendrites may affect the way in which neurons integrate synaptic activity. It is important to consider in the discussion of this manuscript that the spread of excitatory synaptic potentials is greatly affected by the geometry of the dendritic tree. Due to the fact that alcohol consumption affects the morphology, it is reasonable that it also affects the waveform of EPSP.

As alcohol consumption increases further the basal dendrites and the number of dendrites in this part of the neuron. The authors should stimulate selectively the glutamatergic and GABAergic fibers in apical or basal dendrites.

The authors use an intracellular solution based on cesium, they should use a solution of the potassium gluconate-based to measure the fundamental characteristics of the action potential and electrical properties of the pyramidal neurons. What is the value to threshold, first spike latency, instantaneous firing frequency, action potential half width (action potential wave form)?

It is possible that the increase of EPSC frequency is due to an enhancement of presynaptic excitability?

Although it is reasonable the assumption that most anatomically identified synapses and axonal projections correspond to functional units. In this paper the correlation between number of dendritic spine and number of synapses it is no clear. The authors had to use the multiplicity index to estimate the change in connectivity see work Hsia et al 1998.

Was the input resistance was measured once to open the seal, during or at the end of the experiment?

In the patch-clamp experiment, how long is the duration of the dialysis?

In line 410, which means "significant level of dendritic plasticity". Is it structural plasticity? Please clarify

The IPSCs were recorded at 0 mV? What was the reversal potential for Cl-?
